# Locally Acquired Leptospirosis in Expedition Racer, Manitoba, Canada

**DOI:** 10.3201/eid2412.181015

**Published:** 2018-12

**Authors:** Sameer S. Kassim, Antonia Dibernardo, L. Robbin Lindsay, Terence C. Wuerz

**Affiliations:** University of Manitoba, Winnipeg, Manitoba, Canada (S.S. Kassim, T.C. Wuerz);; Public Health Agency of Canada, Winnipeg (A. Dibernardo, L.R. Lindsay);; St. Boniface General Hospital, Winnipeg (T.C. Wuerz)

**Keywords:** leptospirosis, Leptospira interrogans, bacteria, recreation, expedition racer, pneumonia, adventure racing, zoonoses, Manitoba, Canada

## Abstract

Leptospirosis is found worldwide, except in northern regions. We report a case associated with a backcountry adventure race in Manitoba, Canada. Initially, nonspecific symptomatology and diagnostic pitfalls contributed to a delay in identification. Careful attention needs to be paid to exposure to and risk for leptospirosis in northern and temperate climates.

Leptospirosis is a zoonotic disease caused by *Leptospira interrogans* (motile bacterial spirochetes). Human transmission occurs by direct contact with contaminated urine or animals (*1*). The organism has a worldwide distribution outside of polar regions and is common during the rainy season in tropical and temperate climates (*2*). Occupational exposure is a reported risk factor in disease-endemic areas (*3*). Other risk factors are recreational exposure to water in temperate and tropical settings, walking barefoot through surface water, contact with wild rodents, and accidental laboratory exposure (*3*). Cases of leptospirosis have been identified at adventure races (*4**,**5*).

Leptospirosis is not reported nationally in Canada or the United States. In the United States, 150–200 hospital admissions for leptospirosis were reported annually during 1998–2000 (*6*). In Canada, only 25 cases with supportive diagnostic results and 36 confirmed cases overall have been observed by the National Microbiology Laboratory (Winnipeg, Manitoba, Canada), which performs testing nationally (Table). In most instances, it is unclear whether these cases were acquired locally or abroad. We report a case of leptospirosis associated with a backcountry adventure race in Manitoba.

**Table:** Confirmed and suspected cases of leptospirosis, Canada, 2009–2017*

Region and province	No. supportive cases†	No. confirmed cases‡	No. cases/region
Western, including the Prairies			
British Columbia	5	6	20
Alberta	2	4	
Saskatchewan	0	0	
Manitoba	3	0	
Central			
Ontario	9	9	37
Quebec	6	13	
Atlantic			
New Brunswick	0	3	4
Nova Scotia	0	1	
Prince Edward Island	0	0	
Newfoundland and Labrador	0	0	
Overall	25	36	61

*As per Centers for Disease Control and Prevention 2013 case definition (*7*). †*Leptospira* agglutination titer >1:200 but <1:800 by microscopic agglutination test for >1 serum specimens. ‡>4-fold increase in *Leptospira* agglutination titer between acute-phase and convalescent-phase serum samples or *Leptospira* agglutination titer >1:800 by microscopic agglutination test for >1 specimens.

In October 2016, a 20-year-old man came to an emergency department in Winnipeg with a 7-day history of fever, dyspnea, cough, pleuritic chest pain, myalgias, sweats, nausea, vomiting, and diarrhea. These signs and symptoms had improved 1–2 days before admission. Rapid deterioration of the condition of the patient and progressive dyspnea and hypoxia were the reasons for his coming to the emergency department.

A complete blood count showed a leukocyte count of 12.1 × 10^9^ cells/L (reference range 4.5× 10^9^–11 × 10^9^ cells/L), 84% neutrophils, and a platelet count of 34 × 10^9^/L (reference range 140 × 10^9^–440 × 10^9^ /L). Hemoglobin level was within the reference range. Aspartate aminotransferase level was 70 U/L (reference range 10 U/L–32 U/L), alanine aminotransferase level 80 U/L (reference value <30 U/L), alkaline phosphatase level 144 U/L (reference range 30 U/L–120 U/L), total bilirubin level 23 μmol/L (reference range 5 μmol/L–21 μmol/L), and direct bilirubin level 13 μmol/L (reference value <7.0 μmol/L).

Blood smear and culture results were negative for bacteria and fungal elements. The patient had a urea level of 13.6 mmol/L (reference range 2.5 mmol/L–7.1 mmol/L) and a creatinine level of 118 μmol/L (reference range 44 μmol/L–106 μmol/L). Results of urinalysis were within reference limits. Computed tomography of the abdomen and chest showed hepatosplenomegaly, nephromegaly, multifocal and subpleural nodular airspace opacities bilaterally with early cavitation, and a small left pleural effusion. There was no evidence of alveolar hemorrhage by imaging.

Within 12 hours, progressive hypoxemia developed; the patient was intubated and mechanically ventilated. He was given 2 doses of ceftriaxone initially, which was changed to meropenem. The patient also received vancomycin, ciprofloxacin, and liposomal amphotericin B for treatment of possible infection with *Blastomyces gilchristii*. A chest drain was inserted, and 1.5 L of serosanguinous exudative fluid was drained. Culture of pleural and bronchoalveolar fluid did not show pathogenic bacteria, mycobacteria, or fungi; PCR results for common respiratory viruses were negative.

Over the next 10 days, the patient’s condition improved and he was extubated. Antimicrobial drugs were discontinued, amphotericin was replaced by oral itraconazole, and he was discharged. An IgM ELISA for leptospirosis performed 5 days after admission showed a positive result. Microscopic agglutination test (MAT) antibody titers for *L. interrogans* were 1:400 for serovar Australis and 1:100 for serovar Canicola. No appropriate clinical samples were available for retrospective confirmatory PCR testing. Itraconazole was discontinued at follow-up, 20 days after discharge.

Ten weeks after admission, the patient remained clinically asymptomatic, and results of a chest radiograph were uneventful. Serologic analysis showed persistently positive *Leptospira* IgM titers but negative MAT titers for all serovars. Repeat ultrasound of the abdomen showed near resolution of the organomegaly.

We retrospectively identified a possible exposure 10 days before admission: the patient had participated in a 9-hour adventure race in the Whiteshell area of southeastern Manitoba. The patient trekked, biked, and paddled through swamps, bogs, and rivers and reported ingesting murky groundwater at various points during the race.

The Whiteshell area is predominantly nature a conservancy and home of many mammals. Most veterinary studies have focused on prevalence of leptospirosis in wildlife species in specific and limited geographic areas. Leptospirosis has been detected in beavers, coyotes, deer, foxes, opossums, and otters in Canada and is especially prevalent in raccoons and skunks in urban and rural environments (*8**,**9*). There are limited published data on endemic leptospirosis in Canada, but sylvatic exposures have been reported among hunters, trappers, and indigenous peoples; the most northern exposure was identified in the Nunavik region of Quebec (*10*).

We report a case of endemically acquired leptospirosis, detected by using supportive laboratory evidence, from an adventure racer in western Canada. As the popularity of these events increases, clinicians should be vigilant for this life-threatening infection in regions not normally considered endemic for this disease. Early diagnosis of leptospirosis based on clinical features and exposure history in temperate and tropical climates should be considered. For suspected cases, an acute-phase serum sample should be collected for serologic testing by IgM ELISA and MAT. Urine or blood should be collected before treatment for PCR testing, followed by convalescent-phase serologic analysis where applicable to assist diagnosis. In addition, appropriate therapy with intravenous penicillin or ceftriaxone should be initiated empirically for severe disease.
